# Inter-observer variability of cribriform architecture and percent Gleason pattern 4 in prostate cancer: relation to clinical outcome

**DOI:** 10.1007/s00428-020-02902-9

**Published:** 2020-08-20

**Authors:** Margaretha A. van der Slot, Eva Hollemans, Michael A. den Bakker, Robert Hoedemaeker, Mike Kliffen, Leo M. Budel, Natascha N. T. Goemaere, Geert J. L. H. van Leenders

**Affiliations:** 1 Anser Prostate Clinic, Maasstadweg 21, 3079 DZ Rotterdam, The Netherlands; 2 Department of Pathology, Maasstad Hospital, Rotterdam, The Netherlands; 3 Department of Pathology, Erasmus MC University Medical Centre, Rotterdam, The Netherlands; 4 Department of Pathology, Franciscus Gasthuis & Vlietland, Rotterdam, The Netherlands

**Keywords:** Prostate cancer, Grade group, Cribriform, Intraductal, Inter-observer variability

## Abstract

The Grade group is an important parameter for clinical decision-making in prostate cancer. Recently, percent Gleason pattern 4 and presence of invasive cribriform and/or intraductal carcinoma (CR/IDC) have been recognized for their independent predictive value for prostate cancer outcome. There is sparse data on the inter-observer agreement for these pathologic features in practice. Our objectives were to investigate inter-observer variability of percent Gleason pattern and CR/IDC and to relate individual tumour scores to clinical outcome. Our cohort included 80 consecutive radical prostatectomies with a median follow-up 87.1 months (interquartile range 43.3–119.2), of which the slide with largest tumour volume was scored by six pathologists for Grade group (four tiers: 1, 2, 3 and 4/5), percent Gleason pattern 4 (four tiers: 0–25%, 26–50%, 51–75% and 76–100%) and presence of CR/IDC (two tiers: absent, present). The individual assignments were related to post-operative biochemical recurrence (20/80). Inter-observer agreement was substantial (Krippendorff’s *α* 0.626) for assessment of Grade group and moderate for CR/IDC (*α* 0.507) and percent Gleason pattern 4 (*α* 0.551). For each individual pathologist, biochemical recurrence rates incremented by Grade group and presence of CR/IDC, although such relation was less clear for percent Gleason pattern 4. In conclusion, inter-observer agreement for CR/IDC and percent Gleason pattern 4 is lower than for Grade groups, indicating awareness of these features needs further improvement. Grade group and CR/IDC, but not percent Gleason pattern 4 was related to biochemical recurrence for each pathologist, indicating overall validity of individual grade assignments despite inter-observer variability.

## Introduction

Radical prostatectomy (RP) is one of the main treatment modalities for prostate cancer. Despite its oncological efficacy, RP is complicated by urinary incontinence and erectile dysfunction in a significant number of men [1, 2]. Since post-operative complications and oncological outcome are significantly affected by the surgical urologists’ experience, RP procedures are increasingly performed in high-volume expert centres [3–6]. Standardized recording of clinicopathological and outcome parameters is a prerequisite for general quality assessment and research purposes in these centres.

Previous studies have shown moderate-substantial inter-observer variability for prostate cancer grading among pathologists [7–10]. These inter-observer studies are often composed of highly selected case sets, might specifically focus on areas of interest and generally lack correlation with clinical outcome. Recently, percent Gleason pattern 4 and presence of invasive cribriform and intraductal carcinoma (IDC) have been identified as independent pathologic parameters associated with adverse clinical outcome [11, 12]. The International Society of Urological Pathology (ISUP) and World Health Organization (WHO) therefore recommend that these features specifically should be included in prostate cancer biopsy and RP reports [13, 14]. In addition, two proof-of-principle studies have recently shown that incorporation of either percent Gleason pattern 4 and 5 (“integrated quantitative” Gleason score; IQ-Gleason) or invasive cribriform and/or intraductal carcinoma (“cribriform grade”; cGrade) in modified prostate cancer grading schemes results in better prediction of clinical outcome than current grading [12, 15]. Little is known on the inter-observer variability of these novel pathologic features and alternative grading schemes. Therefore, the objectives of this study are to determine the inter-observer variability in prostate cancer grading, percent Gleason patterns, presence of CR/IDC and alternative grading systems in an unselected cohort of RP specimens and to relate individual assessments to biochemical recurrence rates.

## Materials and methods

### Case selection

Consecutive surgical specimens of 80 patients who had undergone RP for prostate cancer in Erasmus MC, University Medical Centre, Rotterdam, The Netherlands, between January 2005 and January 2007 were collected. None of the patients had received radiation or hormonal therapy prior to operation, or before biochemical recurrence was proven. Cases were only included if follow-up was available. Each RP had been transversely sectioned into 4-mm slides from apex to base. Each slide was subsequently cut and embedded in two halves or four quarters depending on its size and routinely processed for haematoxylin and eosin (H&E) staining. All RP slides had previously been reviewed for study purposes by two investigators (EH, GvL) [16]. One investigator (EH) who did not participate in the inter-observer study selected the most representative H&E slide with the largest tumour area and highest Gleason score per case. Both features were represented by the same index tumour in all cases. These 80 slides were digitally scanned using a Hamamatsu Nanozoomer 2.0 HT scanner (Hamamatsu Photonics K.K., Hamamatsu City, Shizuoka Pref., Japan; magnification 40×; pixel size 0.23 μm). The study was approved by the institutional Medical Ethical Committee (MEC-2018-1614).

### Pathologic evaluation

A group of six pathologists with interest in genitourinary pathology from three different medical centres in Rotterdam, The Netherlands, participated in the inter-observer study. Each pathologist evaluated the 80 slides, either as digital image or as actual slide, depending on personal preference. The following parameters were recorded by each pathologist: Gleason score/Grade group according to the 2014 ISUP/2016 WHO guidelines, Gleason pattern 4 and 5 percentage, presence of invasive cribriform and/or intraductal carcinoma (CR/IDC), extra-prostatic extension (EPE) and surgical margin status [14, 17]. For further analyses, we categorized Gleason pattern 4 percentage as 0–25%, 26–50%, 51–75% and 76–100%. Gleason pattern 5 was grouped as being absent, ≤ 5% (tertiary pattern), 6–50% (secondary pattern) and > 50% (primary pattern). Since no immunohistochemical stainings were available in this study, no distinction was made between invasive cribriform and intraductal carcinoma.

### Alternative grading systems

In recent years, prostate cancer grading systems incorporating either percent Gleason pattern 4 and 5 or invasive cribriform and intraductal carcinoma have been proposed [12, 15]. Sauter and colleagues developed an “integrated quantitative” Gleason score (IQ-Gleason), in which the absolute quantitative percentages of any Gleason pattern 4 and 5 were added up. If any pattern 5 is seen, 10 points are added and another 7.5 points if Gleason pattern 5 quantity is larger than 20%. The final score therefore ranges from 0 to 117.5. To allow for comparison with Grade groups, we categorized IQ-Gleason in four groups: 0–25, 26–50, 51–75 and 76–117.5. Another system was proposed by our group as cribriform Grade (cGrade), in which the original Grade group is decreased by one point if no invasive cribriform and intraductal carcinoma is present, while Grade group 1 with any of these growth pattern is assigned cGrade 2 [12].

### Clinical follow-up

Clinical follow-up after RP consisted of six-monthly and later annual monitoring of serum prostate-specific antigen (PSA) levels. Biochemical recurrence was defined as PSA levels ≥ 0.2 ng/ml measured at two consecutive points in time, at least 3 months apart with undetectable PSA levels after operation, or as PSA increase of > 2.0 ng/ml when serum PSA had not declined to zero after operation.

### Statistical analysis

Krippendorff’s *α* was used to estimate inter-observer agreement for pathologic parameter assessment. Grade group, percent Gleason pattern 4 and percent Gleason pattern 5 were included as ordinal measures, while CR/IDC was considered as nominal parameter. Inter-observer agreement was interpreted as follows: ≤ 0.2 poor, 0.21–0.40 fair, 0.41–0.60 moderate, 0.61–0.80 substantial and > 0.80 almost perfect. Individual pathologic scores were related to biochemical recurrence (BCR) rates and visualized using Kaplan-Meier curves. Statistical analyses were performed using R version 3.61.1.

## Results

### Clinicopathologic characteristics

The median age of the 80 men who had consecutively undergone RP was 64.7 years (interquartile range (IQR) 60.3–67.7), and their pre-operative PSA level was 7.7 ng/ml (IQR 4.8–12.0). Median follow-up was 87.1 months (IQR 43.3–119.2), with biochemical recurrence occurring in 20 (25%) patients.

### Inter-observer variability and clinical outcome of Grade groups

**Fig. 1:**
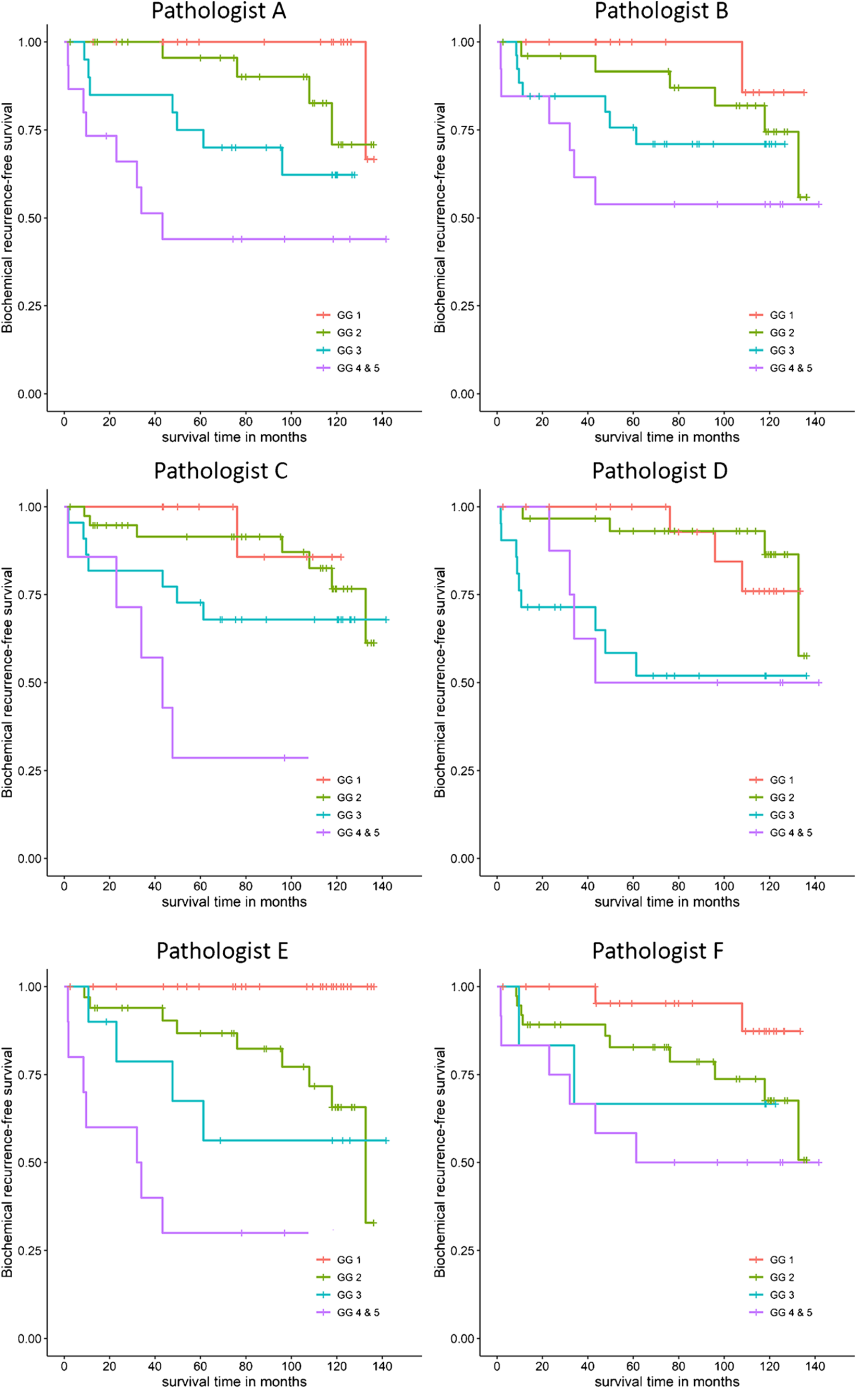
Biochemical recurrence-free survival curves for the different grade groups of each pathologist (GG = grade group)

In 57/80 (71%) cases, at least four out of six pathologists agreed on the Grade group, with complete agreement of all (6/6) pathologists in 8 (10%) cases (Table 1). Grade group assignment was similar or differed by one point in 54 cases (68%). Overall agreement for Grade group was substantial (Krippendorff’s *α* 0.626). In order to determine the Grade groups’ discriminative value for each pathologist, we related the individual outcomes to biochemical recurrence rates. Since total numbers of Grade group 4 and 5 patients were relatively low, we combined both Grade groups. We found that biochemical recurrence incremented with Grade group for each individual pathologist (Table 2; Fig. 1). The Grade group assessment of pathologist E had most discriminative value for biochemical recurrence in this cohort.

**Table 1:** Agreement between observers on assignment of pathologic variables

	Observer agreement					
Pathologic parameter	2/6	3/6	4/6	5/6	6/6	*α*
Grade group	1 (1%)	22 (28%)	29 (36%)	20 (25%)	8 (10%)	0.626
CR/IDC	-	3 (4%)	15 (19%)	22 (28%)	40 (50%)	0.507
Percent pattern 4	8 (10%)	26 (33%)	12 (15%)	13 (16%)	21 (26%)	0.551
Percent pattern 5	-	5 (6%)	13 (16%)	12 (15%)	50 (63%)	0.455
EPE	-	5 (6%)	6 (8%)	12 (15%)	57 (71%)	0.622
Surgical margin	-	6 (8%)	5 (6%)	5 (6%)	64 (80%)	0.526
IQ-Gleason	6 (8%)	25 (31%)	12 (15%)	10 (8%)	27 (34%)	0.597
cGrade	4 (5%)	20 (25%)	25 (31%)	13 (16%)	18 (23%)	0.629

*CR/IDC* invasive cribriform and/or intraductal carcinoma, *EPE* extra-prostatic extension, *α* Krippendorff’s *α*

**Table 2:** Post-operative biochemical recurrence rate for Grade group, invasive cribriform and/or intraductal carcinoma, and percent Gleason pattern 4 per observer

	Observer					
	A	B	C	D	E	F
Grade group						
GG 1	1/18 (6%)	1/15 (7%)	1/12 (8%)	3/21 (14%)	0/27 (0%)	2/25 (8%)
GG 2	4/27 (15%)	6/26 (23%)	7/39 (18%)	4/30 (13%)	9/33 (27%)	10/37 (27%)
GG 3	7/20 (35%)	7/26 (27%)	7/22 (32%)	9/21 (43%)	4/10 (40%)	2/6 (33%)
GG 4/5	8/15 (53%)	6/13 (46%)	5/7 (71%)	4/8 (50%)	7/10 (70%)	6/12 (50%)
Invasive cribriform and/or intraductal carcinoma						
No	12/54 (22%)	10/55 (18%)	9/52 (17%)	9/55 (16%)	10/52 (19%)	9/59 (15%)
Yes	8/26 (31%)	10/25 (40%)	11/28 (39%)	11/25 (44%)	10/28 (36%)	11/21 (52%)
Percentage pattern 4						
0–25%	5/35 (14%)	7/36 (19%)	5/41 (12%)	6/40 (15%)	8/53 (15%)	9/53 (17%)
26–50%	1/12 (8%)	1/10 (10%)	5/12 (42%)	3/16 (19%)	6/13 (46%)	6/17 (35%)
51–75%	6/13 (46%)	3/13 (23%)	3/18 (17%)	6/14 (43%)	4/8 (50%)	3/7 (42%)
76–100%	8/20 (40%)	9/21 (43%)	7/9 (78%)	5/10 (50%)	2/6 (25%)	2/3 (67%)

*GG* grade group

### Invasive cribriform and/or intraductal carcinoma

In 77/80 (96%) cases, at least four out of six pathologists agreed on the presence of invasive CR/IDC, with full agreement in 40 (50%) cases (Table 1). In 31 (39%) cases, all pathologists agreed that CR/IDC was absent, and in 9 (11%), all consented it was present (Krippendorff’s *α* 0.507). For each pathologist, biochemical recurrence occurred more frequently in patients with CR/IDC (Table 2).

### Gleason pattern 4 and 5 percentage

The relative quantity of Gleason pattern 4 was categorized in four groups: 0–25%, 26–50%, 51–75% and 76–100%. In 21 (26%) cases, percent Gleason pattern 4 category was similar for each pathologist, and in 16 (20%) cases, it differed by one point (Krippendorff’s *α* 0.551). While biochemical recurrence rates incremented with higher percent Gleason pattern 4 for pathologist D and F, such relation was less clear for the other pathologists (Table 2). Gleason pattern 5 was grouped as being absent, ≤ 5%, 6–50% and > 50%. In 50 (63%) cases, Gleason pattern 5 subgroup was similar for each pathologist, of which in all cases, it was recorded as absent (Krippendorff’s *α* 0.455). Due to the relatively low number of cases with primary, secondary or tertiary pattern 5, its relation with biochemical recurrence was not further analysed.

### Extra-prostatic extension and surgical margin status

Fifty-seven (71%) cases had complete agreement for extra-prostatic extension, of which eight (10%) were positive and 49 (61%) were negative (Krippendorff’s *α* 0.622; Table 1). In 64 (80%) cases, all pathologists consented on surgical margin status, which was positive in three (4%) and negative in 61 (76%) cases (Krippendorff’s *α* 0.526; Table 1). For each pathologist, biochemical recurrence occurred more frequently in men with extra-prostatic extension and positive surgical margin status (Table 3).

**Table 3:** Post-operative biochemical recurrence rate for extra-prostatic extension and positive surgical margin per observer

	Observer					
	A	B	C	D	E	F
Extra-prostatic extension						
No	8/59 (14%)	10/65 (15%)	10/66 (15%)	10/64 (16%)	10/66 (15%)	8/57 (14%)
Yes	12/21 (57%)	10/15 (67%)	10/14 (71%)	10/16 (63%)	10/14 (71%)	12/23 (52%)
Positive surgical margin						
No	16/70 (23%)	13/69 (19%)	18/74 (24%)	16/73 (22%)	17/71 (24%)	13/66 (20%)
Yes	4/10 (40%)	7/11 (64%)	2/6 (33%)	4/7 (57%)	3/9 (33%)	7/14 (50%)

### Inter-observer variability of IQ-Gleason and cGrade grading schemes

The IQ-Gleason was calculated based on percent Gleason pattern 4 and 5 and categorized in four groups: 0–25, 26–50, 51–75 and 76–117.5. The cGrade was calculated by accounting the original Grade group for the presence of CR/IDC. In 49/80 (61%) cases, at least four out of six pathologists agreed on the IQ-Gleason group (Krippendorff’s *α* 0.597) and 56/80 (70%) on the cGrade (Krippendorff’s *α* 0.629; Table 1). All six pathologists agreed on the IQ-Gleason in 27 (34%) cases and on the cGrade in 18 (23%) cases. For relation with biochemical recurrence, cGrade group 4 and 5 were combined. Biochemical recurrence rates incremented stepwise for the four IQ-Gleason subgroups for pathologist E only, and for the cGrade for pathologist B, D and F (Table 4).

**Table 4:** Post-operative biochemical recurrence rate for alternative IQ-Gleason group and cGrade prostate cancer grading schemes per observer

	Observer					
	A	B	C	D	E	F
IQ-Gleason						
0–25	5/34 (15%)	6/34 (18%)	5/41 (12%)	5/36 (14%)	6/50 (12%)	7/49 (14%)
26–50	0/11 (0%)	1/8 (13%)	2/9 (22%)	2/17 (12%)	4/12 (33%)	5/16 (31%)
51–75	6/12 (50%)	2/10 (20%)	4/18 (22%)	4/10 (40%)	3/7 (43%)	1/5 (20%)
76–117.5	9/23 (39%)	11/28 (39%)	9/12 (75%)	9/17 (53%)	7/11 (64%)	7/10 (70%)
cGrade group						
cGG 1	5/30 (17%)	6/36 (17%)	6/39 (15%)	5/43 (12%)	4/41 (10%)	7/50 (14%)
cGG 2	3/29 (10%)	4/19 (21%)	3/22 (14%)	5/17 (29%)	8/25 (32%)	5/14 (36%)
cGG 3	4/10 (40%)	4/14 (29%)	6/12 (50%)	6/14 (43%)	2/7 (29%)	4/9 (44%)
cGG 4/5	8/11 (73%)	6/11 (55%)	5/7 (71%)	4/6 (67%)	6/7 (86%)	4/7 (57%)

*cGG* cGrade group

## Discussion

Percent Gleason pattern 4 and CR/IDC have been recognized as independent prognostic factors for prostate cancer outcome, but little is known yet on their inter-observer reproducibility in daily clinical practise. In this study, we found moderate inter-observer agreement for CR/IDC (*α* 0.507) and percent Gleason pattern 4 (*α* 0.551) in non-selected RP specimens, which was lower than for Grade groups (*α* 0.626). For the first time, we also investigated the inter-observer variability of two alternative prostate cancer grading models, of which the cGrade (*α* 0.629) had comparable and IQ-Gleason (*α* 0.597) slightly lower reproducibility than conventional Grade groups [12, 15]. Finally, we determined the discriminative value of pathologic parameter assignment for post-operative biochemical recurrence rates for each individual pathologist. Despite inter-observer variability, we found that assignment of Grade groups and CR/IDC were associated with incremental biochemical recurrence rates for each pathologist. Although inter-observer variability of Grade groups has impact on clinical decision-making for individual patients, its effect on population-based studies might be less pronounced. Since WHO and ISUP guidelines recommend reporting of CR/IDC and percent Gleason pattern 4, awareness for their recognition, pathologic delineation and quantification should be raised among the global pathologic community [13, 14].

Many groups have investigated inter-observer variability of Gleason grading over the years and generally showed fair to substantial agreement [7, 8, 10, 18–20]. The set-up of these studies, however, is highly variable and differs in number and experience of participants, in assessment of whole slides or annotated areas, as well as case selection. Amongst 24 international experts in prostate pathology, Egevad et al. for instance found substantial agreement (*κ* 0.67) for grading 90 prostate cancer microphotographs with two-third consensus in 50 (55.6%) cases [9]. Allsbrook et al. investigated inter-observer reproducibility amongst urologic and general pathologists and found higher agreement between urologic pathologists as compared with general pathologists [8, 18]. In our study, six pathologists with interest in genitourinary pathology reached a two-third consensus in 71% of cases with substantial agreement (*α* 0.626), which is well in line with previous studies. A disadvantage of many inter-observer studies is the lack of a gold standard. Some studies include panellists’ consensus diagnosis as reference, but this might result in a bias since ambiguous cases without consensus are excluded. Since we selected consecutive RP specimens with clinical follow-up, we had the opportunity to determine the discriminative value for individual pathologists’ assignments as more objective endpoint. Despite of inter-observer variability, we found that biochemical recurrence rates incremented by Grade group for each participant, indicating the validity of the grade assignment for each individual pathologist.

Although inter-observer variability for Gleason score has been well-studied, little is known yet on agreement for extra-prostatic extension and surgical margin status. Van der Kwast et al. reviewed all slides of 552 radical prostatectomy cases of 11 hospitals and found fair to moderate agreement for extra-prostatic extension (*κ* 0.33) and surgical margin status (*κ* 0.45) [21]. Evans et al. found substantial agreement for extra-prostatic extension (*κ* 0.63) and surgical margin status (*κ* 0.74) at evaluation of 60 slides by 12 expert urologic pathologists [22]. The level of agreement for extra-prostatic extension (*α* 0.622) in our study was comparable with that from Evans et al., but it was lower for surgical margin status (*α* 0.526). For each participant, assessment of extra-prostatic extension and surgical margin status related to biochemical recurrence rates.

Percent Gleason pattern 4, invasive cribriform carcinoma, IDC and tertiary Gleason pattern 5 have all been identified as independent parameters for prostate cancer outcome and should be reported in conjunction with Grade group [13]. Since they affect outcome and might be considered in therapeutic decision-making, quantification and reproducible diagnosis of these recently acknowledged features are increasingly important. To date, only few studies have investigated inter-observer variability of percent pattern 4, invasive cribriform and intraductal carcinoma [20, 23, 24]. Sadimin et al. found substantial agreement of percent Gleason pattern 4 in 422 biopsy cores for one senior genitourinary pathologist and his four fellows [23]. Reproducibility of percent pattern 4 categorized in four subgroups was only moderate in our study. The better performance of the first study can be attributed to the fact the participants had been closely collaborating in common sessions, which contrasts to the situation in our study. In our study, percent Gleason pattern 4 showed stepwise increase of biochemical recurrence rate in only two out of six pathologists. Although this might imply percent Gleason pattern 4 is not a reliable predictor for clinical outcome, this conclusion cannot be drawn from this study, as presence of Gleason pattern 5 was not accounted for. In a study designed for subclassification of Gleason 4 growth pattern among 23 expert genitourinary pathologists, Kweldam et al. found higher level of agreement for cribriform and glomeruloid than for poorly formed and fused glandular structures [20]. Among 337 pathologists, Egevad et al. also revealed poorer reproducibility for poorly formed and fused glands than cribriform architecture [25]. In our unselected cohort of RP, moderate inter-observer variability was observed for identification of CR/IDC. Since previous study revealed that cribriform pattern is more reproducible than poorly formed and fused glands, a higher level of agreement for identification of cribriform and intraductal carcinoma could have been expected [20, 25]. This discrepancy might be caused by the lack of a generally accepted definition of cribriform carcinoma and its distinguishing features from complex fused and glomeruloid structures. Apart from better delineation of both invasive and intraductal carcinoma, further awareness for recognition and reporting of invasive cribriform carcinoma, IDC and percent Gleason pattern 4 within prostate cancer health care should be raised. Griffiths et al. showed an improvement of inter-observer agreement after training, while web-based tutorials could help to improve awareness and decrease inter-observer variability [9, 19].

Two proof-of-principle studies, respectively, incorporating percent Gleason pattern 4/5, or invasive and intraductal carcinoma in novel grading schemes, have shown better discriminative value than current Grade groups. Here, we calculated tumour grade according to the IQ-Gleason and cGrade systems and for the first time analysed the inter-observer variability of these grading schemes. Inter-observer agreement of the cGrade system (*α* 0.629) was comparable with that from the Grade groups (*α* 0.626), while it was slightly lower for IQ-Gleason groups (0.597). It is, however, important to note that IQ-Gleason represents a continuous scale from 0 to 117.5, and that our 4-tier subcategorization for comparison purposes is not advocated for the IQ-Gleason. The comparable reproducibility of the cGrade and Grade group system is explained by the fact that cGrade is based on the Grade group system with adaptation for the presence/absence of CR/IDC. Although numbers are relatively low, we showed that biochemical recurrence was more frequent for cGrade 1 as compared with Grade group 1. cGrade 1 includes all Grade group 1 and 2 tumours without CR/IDC. Recent studies indicate that CR/IDC mostly correlates with the development of lymph node and distant metastasis, and to a lesser extent with biochemical recurrence [12, 26]. The current study cohort was neither developed nor powered for statistical comparison of the three grading schemes, so that general conclusions on their relative performance cannot be drawn.

To our knowledge, this is the first study relating inter-observer variability in prostate cancer grading to occurrence of biochemical recurrence after RP. This objective outcome measure gives unique information on the discriminative value and validity of individual pathologist’s grade assignment. Furthermore, the unselected study cohort of 80 consecutive RP specimens and participation of general pathologists is more representative for clinical practise than cohorts selected after consensus diagnosis by an expert panel, or for the presence of specific features. Finally, we specifically investigated inter-observer variability of recently acknowledged prognostic pathologic features and potential alternative grading schemes. The caveat of this study was the relatively low number of participants, which were selected for their individual participation in a regional prostate cancer network. Although we related individual pathologic assessments to clinical outcome, the number of cases and events was too low for further statistical analysis.

Despite prostate cancer inter-observer variability, individually assigned Grade groups had discriminative value for biochemical recurrence for each pathologist. Although grading variability might significantly affect clinical decision-making for prostate cancer patients, this relation with clinical outcome validates the overall reliability of individual tumour grading. Agreement on recently acknowledged percent Gleason pattern 4, invasive cribriform and intraductal carcinoma was only moderate, stressing the importance of raising widespread awareness and recording of these novel parameters.

## Data Availability

Not applicable.
